# Relative Efficacy of Alirocumab, Evolocumab, Inclisiran, and Bempedoic Acid on Lipids in Patients with Cardiovascular Disease or Familial Hypercholesterolaemia

**DOI:** 10.3390/jcm14227946

**Published:** 2025-11-10

**Authors:** Sophia Khattak, Antonio Ochoa-Ferraro, Nazish Khan, Sudhakar George, Sohail Q. Khan, Jonathan N. Townend, Charlotte Dawson, Mark R. Thomas

**Affiliations:** 1 Institute of Cardiovascular Sciences, University of Birmingham, Edgbaston, Birmingham B15 2TT, UK; s.khattak@bham.ac.uk (S.K.);; 2 Queen Elizabeth Hospital, Mindelsohn Way, Edgbaston, Birmingham B15 2WB, UK; 3 National Institute for Health and Care Research (NIHR) Birmingham Biomedical Research Centre, Birmingham B15 2TH, UK

**Keywords:** lipid lowering therapy, LDL reduction, dyslipidaemia, cholesterol, atherosclerosis

## Abstract

**Background:** Lowering lipid levels after an acute coronary syndrome is critical for preventing recurrent adverse cardiovascular events. Multiple medications are now available, but there is limited evidence comparing how frequently they lead to the achievement of guideline-recommended lipid targets. **Methods and Results:** This observational study evaluated the impact of novel lipid-lowering therapies (alirocumab, evolocumab, inclisiran, and bempedoic acid) in patients with a history of atherosclerotic cardiovascular disease or familial hypercholesterolaemia treated with maximum-tolerated doses of high-intensity statin therapy with or without ezetimibe. Our primary assessment was the achievement of LDL-C below 1.4 mmol/L as per the European Society of Cardiology guidelines. The study comprised of 256 patients. Reduction in LDL-C was greatest with alirocumab and evolocumab, achieving a reduction of 62% (95% confidence interval [CI], 51 to 93; *p* < 0.001) and 58% (95% CI, 47 to 88; *p* < 0.001) after 12 months, respectively. This was followed by inclisiran with a reduction of 47% (95% CI, 37 to 78; *p* < 0.001) and bempedoic acid with a reduction of 36% (95% CI, 22 to 69; *p* < 0.001). Patients treated with alirocumab and evolocumab started from a higher baseline LDL-C than inclisiran, due to the higher LDL threshold required for initiation of alirocumab and evolocumab in the UK. Despite this, inclisiran, evolocumab, and alirocumab were all associated with similar proportions of patients achieving LDL targets: 35%, 42%, and 37% of patients achieved a guideline-recommended LDL-C target of <1.4 mmol/L. Patients with a baseline LDL-C > 4 mmol/L were more likely to reach the ESC target when treated with alirocumab or evolocumab compared to inclisiran, with results of 33.3% vs. 24.1% (*p* = 0.016) and 35.7% vs. 24.1% (*p* = 0.05). **Conclusions:** Alirocumab and evolocumab were associated with the greatest reductions in LDL-C, followed by inclisiran and bempedoic acid. Overall, alirocumab, evolocumab, and inclisiran led to approximately 40% of patients reaching ESC targets for LDL-C. In patients with a baseline LDL-C > 4 mmol/L, significantly more patients achieved LDL-C targets when treated with alirocumab or evolocumab compared to inclisiran. **Strength and limitations:** This was the first study to comprehensively compare the efficacy of novel lipid-lowering therapies in achieving guideline-recommended LDL targets within a high-risk cardiovascular population. The sample size was relatively small, especially for patients treated with bempedoic acid.

## 1. Introduction

Cardiovascular disease (CVD) is a major health burden, causing significant morbidity and mortality worldwide. High plasma lipid levels, especially low-density lipoprotein cholesterol (LDL-C), play a major role in the development and progression of atherosclerotic cardiovascular disease. Lipid-lowering therapies are therefore critical in preventing and treating coronary artery disease [1,2]. Statins and ezetimibe have been highly effective in reducing LDL-C levels and preventing cardiovascular events in patients with coronary artery disease. However, despite therapy with statins and ezetimibe, some patients still have suboptimal LDL-C levels as a result of either limited efficacy or tolerability, leading to residual CVD risk and resulting in preventable cardiovascular events. This has necessitated the development of additional lipid-lowering therapies, such as PCSK9 inhibitors (alirocumab and evolocumab), inclisiran, and bempedoic acid [2,3,4]. These medications differ in their mechanisms of action and in the extent of LDL cholesterol reduction that they achieve.

The currently approved PCSK9 inhibitors are monoclonal antibodies that inhibit PCSK9-mediated LDL receptor degradation, resulting in increased hepatic clearance of LDL-C [5]. Trials have shown an approximately 50–60% reduction in LDL-C levels and 15% reduction in the risk of major adverse cardiovascular events when PCSK9 inhibitors are added to statin therapy in patients with previous MI [6,7,8]. Inclisiran is a first-in-class small interfering RNA that also targets the PCSK9 pathway by inhibiting the production of PCSK9 [9]. The ORION trials have shown up to 50% reduction in LDL-C levels and clinical outcome studies are awaited [10]. Bempedoic acid inhibits adenosine triphosphate citrate lyase in the liver, thus reducing hepatic LDL-C synthesis. The CLEAR harmony trial showed a reduction in LDL-C of approximately 18% as an adjunct to statin therapy and the CLEAR OUTCOMES study showed a 13% reduction in major adverse cardiovascular events in statin-intolerant patients with a high risk of cardiovascular disease [11,12].

There are now several different possible options for lowering LDL-C after acute coronary syndromes when statins are ineffective or not tolerated, but there is little evidence to help choose the optimal strategy. Although clinical trials of the individual medications have shown their impact on LDL-C, these clinical trials differ in their study designs and populations. Many of the populations studied are also not representative of diverse populations encountered in clinical practice. We therefore investigated whether different lipid-lowering treatments in a high-risk population were associated with different rates of achieving guideline-recommended lipid targets in routine clinical practice in the UK.

## 2. Methods

### 2.1. Study Design, Setting, and Participants

In this observational study, we identified all consecutive eligible patients treated in the Lipid and Preventive Cardiology Clinic at University Hospitals Birmingham database. Patients were referred from primary care, cardiology, or other hospital specialties for assessment and management of dyslipidaemia. The clinic, developed from a pre-existing lipid service, is a recently established state-of-the-art multi-disciplinary centre serving a diverse, multi-ethnic population with high levels of socioeconomic deprivation. Treatment with PCSK9 inhibitors was initiated between October 2017 and January 2024, while other therapies were started between November 2020 and March 2024.

Patients were eligible for inclusion if they had atherosclerotic cardiovascular disease demonstrated on imaging, a previous acute coronary syndrome, or familial hypercholesterolaemia, and were initiated on one of the following novel lipid-lowering therapies: alirocumab, evolocumab, inclisiran, or bempedoic acid. Patients were excluded if they had changes in background lipid-lowering therapy during follow-up, or if they were initiated on more than one novel lipid-lowering medication, as this would preclude interpretation of the independent effects of each agent. All patients meeting inclusion criteria during this period were consecutively included without random sampling. Data were extracted retrospectively from electronic medical records.

At baseline, all patients were on the highest tolerated dose of high-intensity statin therapy (atorvastatin 20–80 mg or rosuvastatin 10–40 mg), or no statin if intolerant, with or without ezetimibe, but had not yet been started on any other lipid-lowering treatment. LDL cholesterol and other parameters were assessed before and after initiation of therapy at 3–6, 6–12, and 12–24 month intervals, reflecting intervals at which LDL-C is checked in routine clinical follow-up.

### 2.2. Lipid Assessments

Baseline characteristics, such as sex, age, body mass index (BMI), comorbidities, and baseline lipid levels, were collected from the electronic healthcare record. The main assessment was the percentage of patients achieving an LDL-C of <1.4 mmol/L as per ESC guidelines [13]. Secondary assessments included percentage of patients achieving <2.0 mmol/L as per the most recent NICE guidelines [14] and absolute and percentage change in levels of LDL-C, total cholesterol, triglycerides, and C-reactive protein. Additionally, we assessed the difference in LDL-C reduction between PCSK-9 inhibitors and inclisiran, both in terms of percentage reduction and evaluating which treatment was more likely to lead to achieving ESC and NICE targets.

### 2.3. Time Points and Follow-Up

As this was a retrospective study, follow-up blood tests were performed at variable intervals depending on routine clinical practice. To account for this variability and ensure adequate sample representation, time intervals (e.g., 3–5.9, 6–11.9, and 12–24 months) were defined rather than fixed time points and the first value was used from each time range. These intervals capture a clinically meaningful period over which changes in LDL-C levels would be expected to occur.

### 2.4. Ethics

The local clinical governance committee (CARMS 21072 IG1029) approved this study as a service evaluation. The National Health Service Health Research Authority decision tool indicated that NHS ethics approval and individual patient consent was not required.

### 2.5. Statistical Analysis

Continuous data are expressed as the mean with a 95% confidence interval (CI). Normally distributed data were compared using paired *t*-tests or ANOVA as appropriate, the Holm–Bonferroni method to correct for multiple comparisons, and Tukey’s Honest Significant Difference (HSD) test for pairwise comparisons. Continuous non-normally distributed variables were compared using the Mann–Whitney U test. Patients’ characteristics were compared using the chi-square test (or Fisher’s exact test for smaller sample sizes) for categorical variables and the Kruskal–Wallis test for continuous variables.

This observational study included all eligible patients during the study period; no formal sample size calculation was performed in advance. Based on the observed variability in baseline LDL-C between groups (Cohen’s f indicating a large effect) and within-group standard deviation, a one-way ANOVA comparing the four treatment groups had over 80% power with substantially fewer than 30 patients per group at an alpha level of 0.05. As each group in our study included at least 30 patients, the analysis was adequately powered for overall between-group comparisons. Effect estimates with 95% confidence intervals are reported, and pairwise post hoc comparisons were adjusted for multiple testing. Statistical analysis was carried out using Jupyter Notebook 6.5.5 in Python version 3.8.18 (Python Software Foundation, Wilmington, DE, USA) using the following packages: Pandas 1.5.3, numpy 1.24.4, scipy 1.10.1 tableone 0.7.14, matplotlib 3.7.2, and seaborn 0.12.2.

## 3. Results

### 3.1. Patient Characteristics

The study comprised a total of 256 patients, of whom 108 received inclisiran, 68 patients received evolocumab, 50 patients received alirocumab, and 30 patients received bempedoic acid. There were minor differences in the mean age and weight between the groups (see Table 1). The study population predominantly comprised older adults with cardiovascular risk factors and elevated BMI, consistent with a high-risk secondary prevention cohort typically referred to specialist lipid and preventive cardiology clinics. This reflects the routine patient profile encountered in tertiary care, where metabolic comorbidities such as obesity, diabetes, and hypertension are common. Patients were most likely to be female (80%) in the bempedoic acid group, likely reflecting the higher prevalence of statin intolerance in females [15]. Comorbidities such as diabetes, hypertension, chronic kidney disease, cerebrovascular disease, and stroke were comparable across groups. Patients starting on alirocumab and evolocumab less commonly had statin intolerance. Alirocumab and evolocumab treated patients had higher baseline LDL-C levels compared to the other groups, reflecting the thresholds required for initiation of these treatments in the UK (threshold LDL-C > 3.5 mmol/L if very high risk of further CVD, threshold LDL-C > 4 mmol/L if high risk of further CVD, or threshold of LDL > 5 mmol/L if familial hypercholesterolaemia without established CVD) [16,17]. The overall tolerability of injectable lipid-lowering therapies was favourable, with 12% discontinuing inclisiran and 16% discontinuing PCSK-9 inhibitors within 2 years.

### 3.2. Reduction in LDL-C

**Figure 1:**
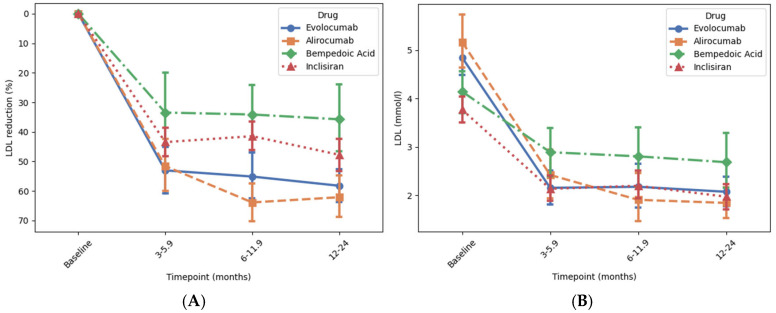
Association of novel lipid-lowering medications with reduction in LDL cholesterol at different time points. Time intervals were used to account for variation in timing of follow-up blood tests in this retrospective study. Panels (**A**) and (**B**) show the percentage change and the absolute reduction in low-density LDL-C level over time, respectively. Change in LDL-C levels from baseline to 12–24 months was analysed using ANOVA followed by post hoc correction for multiple comparisons. There was a significant reduction in LDL-C with all drugs (all *p* < 0.001) (Supplementary Table S1). Error bars represent 95% confidence interval around the mean.

**Figure 2:**
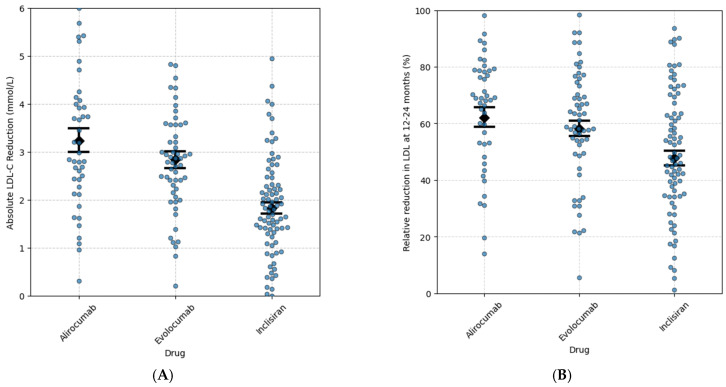
Absolute (**A**) and relative (**B**) reduction in LDL-C associated with alirocumab, evolocumab, and inclisiran at 12–24 months. The monoclonal PCSK9 inhibitors alirocumab and evolocumab had a similar LDL-C percentage reduction at 12–24 months (Tukey HSC alirocumab vs. evolocumab = 0.682), whereas there was a greater LDL-C % reduction with the monoclonal PCSK-9 inhibitors at 12–24 months compared with inclisiran (Tukey HSD, alirocumab vs. inclirisan = 0.003 and evolocumab vs. inclisiran. = 0.027). Error bars represent 95% confidence interval around the mean. Each dot represents an individual patient.

Bempedoic acid, inclisiran, and the PCSK9 inhibitors were all associated with a reduction in LDL-C compared to baseline (all *p* < 0.001), which was achieved within the first 3 months and was sustained until 12–24 months in each case (Figure 1). The greatest reduction in LDL-C was achieved in patients treated with alirocumab and evolocumab, which was a significantly greater reduction than achieved in patients treated with inclisiran or bempedoic acid (both *p* < 0.05, Figure 2).

Patients treated with alirocumab or evolocumab both had a mean baseline LDL-C of approx. 5 mmol/L, which decreased by approx. 60% compared to baseline (*p* < 0.001 for both) to a mean of approx. 2 mmol/L after 12–24 months, with approx. 40% of patients reaching the ESC target (LDL-C below 1.4 mmol/L) and approx. 60% reaching the NICE target (LDL-C below 2.0 mmol/L). Patients treated with inclisiran and bempeodic acid both had a lower mean baseline of approx. 4 mmol/L. In patients treated with inclisiran, LDL-C decreased by 48% compared to baseline (*p* < 0.001) to a mean of 1.97 mmol/L after 12–24 months with 35% or patients reaching the ESC target and 58% reaching the NICE target. In patients treated with bempedoic acid, LDL-C decreased by 36% compared to baseline (*p* < 0.001) to a mean of 2.68 mmol/L after 12–24 months, with 12% of patients reaching the ESC target and 29% reaching the NICE target.

We also specifically compared the absolute LDL-C reduction at 12–24 months among patients treated with the injectable LDL-C-lowering medications, alirocumab, evolocumab, and inclisiran Figure 2A. Patients treated with alirocumab or evolocumab had a greater LDL-C reduction than those treated with inclisiran (mean difference 1.01 mmol/L, 95% CI 0.49 to 1.52, *p* < 0.05, and mean difference 1.41 mmol/L, 95% CI 0.84 to 1.98, *p* < 0.05, respectively) Figure 2A. No statistically significant difference was observed between evolocumab and alirocumab (mean difference 0.4 mmol/L; 95% CI: −0.21 to 1.00; *p* = 0.27) Figure 2A.

Inclisiran, evolocumab, and alirocumab were associated with a similar percentage of patients achieving the ESC target of LDL-C < 1.4 mmol/L (35%, 42%, and 37%, respectively). Similar percentages of patients also achieved the NICE target of LDL-C < 2.0 mmol (58%, 66%, and 56%, respectively). Bempedoic acid was associated with a significantly lower percentage of patients achieving the ESC target of <1.4 mmol/L (12%) or the NICE target of <2.0 mmol/L (29%). When comparing patients on high-intensity statin doses to those on lower doses or no statin, there were similar changes in LDL-C in each group. However, an exception was observed with bempedoic acid, where greater LDL reduction was seen without a high-intensity statin (See Supplementary Figure S1).

**Figure 3:**
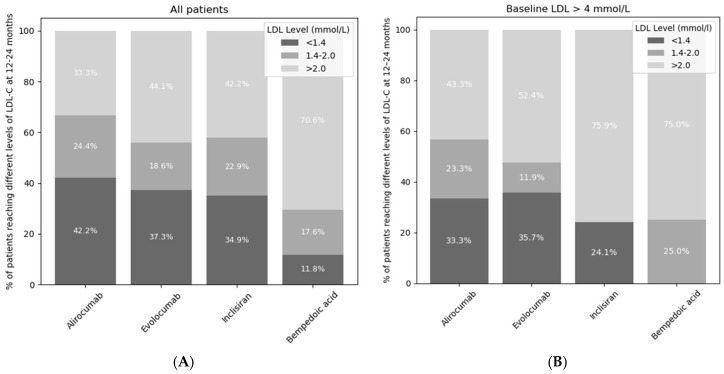
Percentage of patients not reaching ESC or NICE targets (light grey), reaching the NICE target but not the ESC target (medium grey), and reaching both the ESC and NICE targets (dark grey) at 12–24 months. The LDL-C targets from ESC guidelines were LDL-C < 1.4 mmol/L and NICE guidelines were <2.0 mmol/L. The darkest grey segments represent patients achieving both ESC and NICE LDL-C targets. (**A**) describes all patients whereas (**B**) only describes patients with baseline LDL > 4 mmol/L.

Patients with a baseline LDL-C over 4 mmol/L were eligible for all of the different LDL-lowering medications, including PCSK9 inhibitors, inclisiran, and bempedoic acid. For patients with an LDL-C of greater than 4 mmol/L at baseline, a greater proportion of patients achieved an LDL-C of <1.4 mmol/L if treated with alirocumab or evolocumab compared to inclisiran (33.3% vs. 24.1%, *p* = 0.016, and 35.7 vs. 24.1%, *p* = 0.051, respectively, Figure 3B).

### 3.3. Reduction in Total Cholesterol

**Figure 4:**
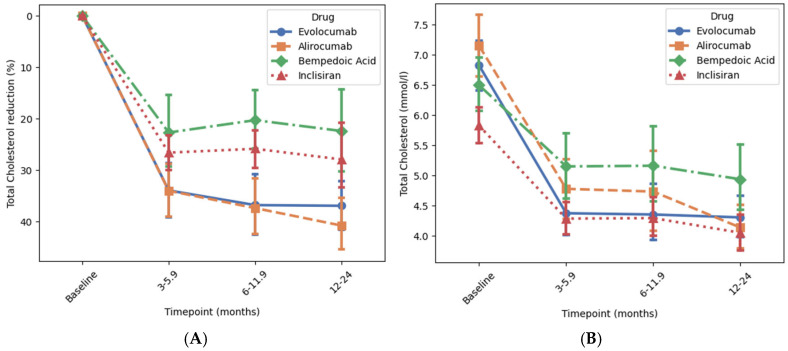
Association of novel lipid-lowering medications with reductions in total cholesterol at different time points. Panels (**A**) and (**B**) show the percentage change and the achieved reduction in total cholesterol level over time, respectively. Change in total cholesterol level from baseline to 12–24 months was analysed using ANOVA followed by post hoc correction for multiple comparisons. *p*-values for the percentage change was <0.001 for all drugs. Error bars represent 95% confidence interval around the mean.

Bempedoic acid, inclisiran, and the PCSK9 inhibitors were also associated with a reduction in total cholesterol (all *p* < 0.001), which was also achieved within the first 3 months in each case Figure 4. Patients treated with alirocumab or evolocumab both had a mean baseline total cholesterol of approx. 7 mmol/L, which decreased by approx. 40% compared to the baseline (*p* < 0.001 for both) to a mean of approx. 4 mmol/L after 12–24 months Figure 4 and Supplementary Figure S2. Patients treated with inclisiran had a baseline total cholesterol of 5.86 mmol/L which reduced by 32% compared to the baseline (*p* < 0.001) after 12–24 months. Patients treated with bempedoic acid had a baseline total cholesterol of 6.5 mmol/L which reduced by 22.3% compared to the baseline (*p* < 0.001) after 12–24 months.

### 3.4. Reduction in Triglycerides and CRP

None of the medications were associated with a significant reduction in triglycerides (Supplementary Table S3 and Figure S2). The percentage and absolute changes in CRP level from baseline to >3 months were also not significant for any of the novel lipid-lowering medications (Table S4, Figure S3).

## 4. Discussion

The primary aim of the study was to assess the proportion of patients with cardiovascular disease that achieve guideline-recommended LDL-C targets in a routine clinical setting. The main findings of this study are as follows: (1). The ESC target of LDL < 1.4 mmol/L was achieved with alirocumab, evolocumab, or inclisiran in approximately 40% of patients. In contrast, a significantly lower proportion of patients receiving bempedoic acid reached this target. (2). Monoclonal PCSK9 inhibitors were associated with greater reductions in LDL-C than inclisiran. (3). Among patients with high baseline LDL-C (>4 mmol/L), those treated with monoclonal PCSK-9 inhibitors were more likely to achieve guideline-recommended LDL-C targets compared to those receiving inclisiran.

In this study, alirocumab, evolocumab, inclisiran, and bempedoic acid were all effective in reducing levels of LDL-C in patients with atherosclerotic cardiovascular disease or familial hypercholesterolaemia who were already treated with maximum-tolerated high dose statin therapy, with or without ezetimibe. The percentage reduction in LDL-C was greatest with the monoclonal PCSK9 inhibitors, alirocumab, and evolocumab, achieving approximately 60% reduction in LDL-C, consistent with the FOURIER and ODYSSEY OUTCOMES trials [6,7], other studies showing similar results [18,19,20]. Inclisiran was associated with a reduction in LDL-C of approximately 50%, in keeping with the findings of the ORION-10 and ORION-11 studies. Patients treated with alirocumab, evolocumab, or inclisiran had an average baseline LDL-C of approximately 4–5 mmol/L. Despite this, approximately 40% reached the ESC LDL-C target of less than 1.4 mmol/L, which compares favourably with previous studies showing that only approximately 20% of patients reach this target when treated with high-intensity statins alone, even when they have a much lower baseline LDL-C [21]. The inhibitory effect of the drugs on LDL-C and total cholesterol was observed within 3–6 months of initiating treatment and then stayed relatively stable until the 12–24 month period. This pattern suggests that the therapies provide early and sustained lipid control and therefore potentially an early reduction in cardiovascular risk [22].

Although they are all mediated by PCSK9, alirocumab and evolocumab have a different mechanism of action to inclisiran and it is not currently well established why inclisiran achieves a lower level of LDL-C reduction. Inclisiran reduces plasma PCSK9 levels by about 80%, possibly indicating less inhibition of PCSK9 than monoclonal antibodies [23]. Monoclonal antibodies, such as alirocumab and evolocumab, also have a broader inhibition and bind to and neutralise PCSK9 produced by hepatocytes, endothelial cells, and macrophages [24]. Inclisiran, in contrast, only silences hepatic PCSK9 mRNA but not PCSK9 mRNA elsewhere [25]. This selective action may result in overall less PCSK9 inhibition compared to the broader inhibition by the monoclonal antibodies. It is also possible that inclisiran’s more infrequent dosing (after 3 months initially and then 6 monthly thereafter) may also contribute, leading to less sustained suppression of PCSK9.

Our study showed that bempedoic acid reduced plasma LDL-C levels by approximately 35%, a greater reduction than the 18% reported in clinical trials [12,26]. This difference may be attributed to differences in our patient population compared to the CLEAR OUTCOMES study, such as the higher proportion of female patients, who seem to derive a disproportionate decrease in LDL-C compared to men [27]. Recent mechanistic studies further support this clinical observation; for instance, Acyl-CoA synthetase-1 (ACSL1), an enzyme pivotal for linking long-chain fatty acid uptake to intracellular metabolic pathways, functions differently in male and female myocardium, indicating fundamental sex-specific differences in lipid metabolism [28]. Despite this decrease in LDL-C, only 30% and 10% of patients, respectively, achieved the ESC- and NICE-recommended LDL-C targets on bempedoic acid. This was largely due to the high baseline LDL-C in patients treated with bempedoic acid, with a mean over 4 mmol/L. Therefore, a mean reduction in LDL-C of 35% was insufficient to achieve either target, raising the possibility of lower clinical efficacy than the other agents.

Alirocumab, bempedoic acid, evolocumab, and inclisiran were not associated with a significant reduction in triglycerides, although it has previously been shown that monoclonal PCSK9 inhibitors cause a modest reduction in levels of triglycerides of approximately 15% [29]. Similarly, we did not demonstrate any changes in levels of CRP, likely because PCSK9 inhibitors and bempedoic acid only cause small reductions in CRP [30]

Overall, our findings suggest that the initiation of alirocumab, evolocumab, and inclisiran were all moderately effective in achieving LDL-C targets. In clinical practice, the choice of which of these to use largely depends on the baseline LDL-C, patient preference, priority of cardiovascular risk reduction, dosing convenience, and cost considerations. Bempedoic acid was associated with the achievement of LDL-C targets less frequently than alirocumab, evolocumab, and inclisiran. In the UK setting, the initiation of bempedoic acid is therefore largely reserved for patients who are not tolerant of statins and not eligible for alirocumab, evolocumab, or inclisiran, or as an adjunct therapy where LDL-C targets have not been achieved with these medications.

## 5. Limitations

Our study has important limitations. This was a retrospective study with the exclusion of patients who were initiated on more than one novel lipid-lowering medication during the follow-up period, as otherwise it would not be possible to interpret the separate effects of each medication. The sample size was relatively small for patients treated with bempedoic acid but was sufficiently powered to detect a 30% reduction in LDL-C. We focused on changes in LDL-C, but larger sample sizes and longer treatment times would be required for the study to be sufficiently powered to assess the impact on clinical outcomes. Another limitation is that we did not have data on statin-related adverse effects or intolerance. Statin-associated muscle symptoms and other side effects are well recognised and represent a leading cause of treatment discontinuation or dose reduction [31], which may have influenced treatment selection and lipid outcomes in this real-world cohort.

## 6. Conclusions

In conclusion, our real-world study demonstrated that monoclonal PCSK9 inhibitors and inclisiran lead to the achievement of the ESC LDL-C target of less than 1.4 mmol/L in less than half of patients, and only 12% of patients treated with bempedoic acid achieved this target. Monocloncal PCSK9 inhibitors were associated with greater reduction in LDL-C than inclisiran and were more likely to lead to patients achieving an LDL-C < 1.4 mmol/L if the baseline LDL-C was over 4 mmol/L. These figures suggest that there is still a need for further lipid-lowering drug development.

## Figures and Tables

**Table 1:** Demographic and clinical characteristics of the patients at baseline (intention-to-treat population).

	Total	Alirocumab	Evolocumab	Inclisiran	Bempedoic Acid	*p*-Value
**Number of patients (% of total)**	**256**	50 (19.5)	68 (26.5)	108 (42.2)	30 (11.7)	
**S** **ex, N (%)**	**F**	18 (36.0)	34 (50.0)	36 (33.3)	24 (80.0)	<0.001
	**M**	32 (64)	34 (50.0)	72 (66.7)	6 (20.0)	
**Age, median** **(Q1, Q3)**		65 [56.0, 73.0]	60.5 [50.5, 67.2]	67.0 [58.2, 72.2]	67.5 [59.2, 74.8]	0.03
**Weight, median (Q1, Q3)**		79.8 [72.1, 92.2]	83.8 [69.4, 93.0]	78.2 [69.0, 92.1]	71 (62.4, 83.7)	0.028
**BMI**		28.3 [26.4, 31.4]	30.0 [25.1, 33.9]	28.1 [24.6, 31.8]	27.2 [25.3, 31.5]	0.318
**E** **thnicity, N (%)**	**Asian**	8 (19.5)	14 (23.3)	20 (20.4)	7 (25.9)	0.650
	**White**	30 (73.2)	41 (68.3)	69 (70.4)	18 (66.7)	
	**Black**	3 (7.3)	4 (6.7)	8 (8.2)	1 (3.7)	
	**Chinese**	0	0	0	1 (3.7)	
	**Other**	0	1 (1.7)	1 (1)	0	
**T1DM, N (%)**		1 (2.0)	2 (3.0)	0	0	0.279
**T2DM, N (%)**		12 (24.0)	16 (24.2)	24 (22.4)	5 (16.7)	0.858
**HTN, N (%)**		26 (52.0)	23 (35.9)	51 (47.7)	9 (30.0)	0.115
**S** **moking, N (%)**	**Ex-smoker**	6 (75.0)	4 (44.4)	18 (52.9)	2 (66.7)	0.738
	**Current smoker**	1 (12.5)	1 (11.1)	7 (20.6)	0	
**CKD, N (%)**		6 (12.0)	4 (6.1)	3 (2.8)	2 (6.7)	0.157
**PVD, N (%)**		1 (2.90)	3 (4.5)	2 (1.9)	0	0.528
**S** **troke, N (%)**		3 (6.0)	1 (1.5)	6 (5.6)	0	0.309
**CABG, N (%)**		8 (16)	6 (9.1)	18 (16.8)	1 (3.3)	0.162
**FH, N (%)**		18 (34)	23 (32.9)	16 (14.7)	10 (31.2)	0.005
**ACS, N (%)**		19 (38)	25 (36.8)	40 (37)	1 (3.3)	0.003
**ACS** **type (%)**	**Stemi**	9 (47.4)	6 (23.1)	15 (35.7)	1 (100)	0.175
	**Nstemi**	11 (55.0)	18 (69.2)	27 (64.3)	0	
	**UA**	0	2 (7.7)	0	0	
**H** **igh intensty statin, N (%)**		21 (77.8)	27 (69.2)	55 (75.3)	5 (22.7)	0.002
**E** **zetimibe, N (%)**		11 (40.7)	21 (52.5)	25 (34.2)	16 (72.7)	0.010
**B** **aseline LDL-C (SD)**		5.2 (2.0)	4.8 (1.6)	3.8 (1.4)	4.1 (1.2)	<0.001
**B** **aseline TG (SD)**		2.1 (1.0)	2.6 (1.9)	2.0 (1.7)	2.2 (1.3)	0.157
**B** **aseline CRP (SD)**		3.9 (2.1)	6.5 (4.3)	3.0 (3.5)	2.3 (1.9)	0.021

*p*-values represent comparisons across all five groups (alirocumab, evolocumab, inclisiran, bempedoic acid, and control) using one-way ANOVA for continuous variables and chi-square or Fisher’s exact test for categorical variables. UA = Unstable angina; T1DM = Type 1 diabetes mellitus; T2DM = Type 2 diabetes mellitus; HTN = Hypertension; CKD = Chronic kidney disease; CABG = Coronary artery bypass graft; FH = Familial hypercholesterolaemia; ACS = Acute coronary syndrome; LDL = Low-density lipoprotein; TG = Triglycerides.

## Data Availability

The original contributions presented in this study are included in the article/Supplementary Material. Further inquiries can be directed to the corresponding author.

## Supplementary Materials

The following supporting information can be downloaded at https://www.mdpi.com/article/10.3390/jcm14227946/s1, Figure S1: Percentage of patients (with or without high intensity dose statin) reaching LDL-C targets as per NICE guidelines (<2.0 mmol/L) and ESC guidelines (<1.4 mmol/L) during treatment with novel lipid-lowering medications at 12–24 months; Figure S2: Association of novel lipid lowering medications with reductions in triglycerides at different time points; Figure S3: Association of novel lipid-lowering medications with changes in CRP at baseline and >3 months; Table S1: LDL-C reduction associated with alirocumab, evolocumab, bempedoic acid and inclisiran at 12–24 months and also averaged across all post-treatment timepoints; Table S2: Total cholesterol reduction associated with alirocumab, evolocumab, bempedoic acid and inclisiran at 12–24 months and also averaged across all post-treatment timepoints; Table S3: Triglyceride reduction associated with alirocumab, evolocumab, bempedoic acid and inclisiran at 12–24 months and averaged across all post-treatment timepoints; Table S4: CRP reduction associated with alirocumab, evolocumab, bempedoic acid and inclisiran at 12–24 months and averaged across all post-treatment timepoints.
