# Supplementary material for: Relative Efficacy of Alirocumab, Evolocumab, Inclisiran, and Bempedoic Acid on Lipids in Patients with Cardiovascular Disease or Familial Hypercholesterolaemia
Source: J Clin Med. 2025 Nov 10;14(22):7946. doi: 10.3390/jcm14227946 (PMC12653900; doi:10.3390/jcm14227946)
Supplement: Supplementary file 1 [file jcm-14-07946-s001.zip › jcm-3910375-supplementary.pdf]

Supplementary Appendix

Relative efficacy of alirocumab, evolocumab, inclisiran, and bempedoic acid on lipids in patients with cardiovascular disease or familial hypercholesterolaemia

Sophia Khattak[1,2], Antonio Ochoa-Ferraro[2], Nazish Khan[1], Sudhakar George[2], Sohail Khan[1,2], Jonathan N Townend[1,2], Charlotte Dawson[2], Mark R. Thomas[1,2].

[1] Institute of Cardiovascular Sciences, University of Birmingham, UK.

[2] Queen Elizabeth Hospital, Birmingham, UK.

Supplementary Figures and Tables

Table S1. LDL-C reduction associated with alirocumab, evolocumab, bempedoic acid and inclisiran at 12-24 months and also averaged across all post-treatment timepoints. P-value calculated using one-way ANOVA.

	DRUG	P-VALUE	BASELINE (MMOL/L)	MEAN ABSOLUTE LDL REDUCTION 12 – 24 MONTHS (MMOL/L)	MEAN POST TREATMENT REDUCTION (MMOL/L)
1	Alirocumab	< 0.001	5.16	3.32	2.83
2	Evolocumab	< 0.001	4.84	2.77	2.81
3	Bempedoic acid	< 0.001	4.14	1.46	3.13
4	Inclisiran	< 0.001	3.76	1.79	2.51

Table S2. Total cholesterol reduction associated with alirocumab, evolocumab, bempedoic acid and inclisiran at 12-24 months and also averaged across all post-treatment timepoints. P-value calculated using one-way ANOVA.

	DRUG	P-VALUE	BASELINE (MMOL/L)	MEAN ABSOLUTE TC REDUCTION 12 – 24 MONTHS (MMOL/L)	MEAN POST TREATMENT REDUCTION (MMOL/L)
1	Alirocumab	<0.001	7.12	2.95	5.06
2	Evolocumab	<0.001	7.03	2.67	5.08
3	Bempedoic acid	<0.001	6.5	1.55	5.44
4	Inclisiran	<0.001	5.86	1.90	4.59

Table S3. Triglyceride reduction associated with alirocumab, evolocumab, bempedoic acid and inclisiran at 12-24 months and averaged across all post-treatment timepoints. P-value calculated using one-way ANOVA.

	DRUG	P-VALUE	BASELINE (MMOL/L)	MEAN ABSOLUTE REDUCTION (MMOL/L)	MEAN POST TREATMENT REDUCTION (MMOL/L)
1	Alirocumab	0.42	2.09	0.32	1.95
2	Evolocumab	0.38	2.61	0.6	2.28
3	Bempedoic acid	0.34	2.17	0.06	2.15
4	Inclisiran	0.49	2.00	0.38	1.77

Table S4. CRP reduction associated with alirocumab, evolocumab, bempedoic acid and inclisiran at 12-24 months and averaged across all post-treatment timepoints. P-value calculated using one-way ANOVA.

	DRUG	P-VALUE	BASELINE (MMOL/L/DL)	MEAN ABSOLUTE REDUCTION (MMOL/L)	MEAN POST TREATMENT REDUCTION (MMOL/L)
1	Alirocumab	0.34	3.85	0.85	3.42
2	Evolocumab	0.14	6.5	1.71	5.64
3	Bempedoic acid	0.53	2.33	-0.33	2.5
4	Inclisiran	0.182	3.02	-0.17	3.11

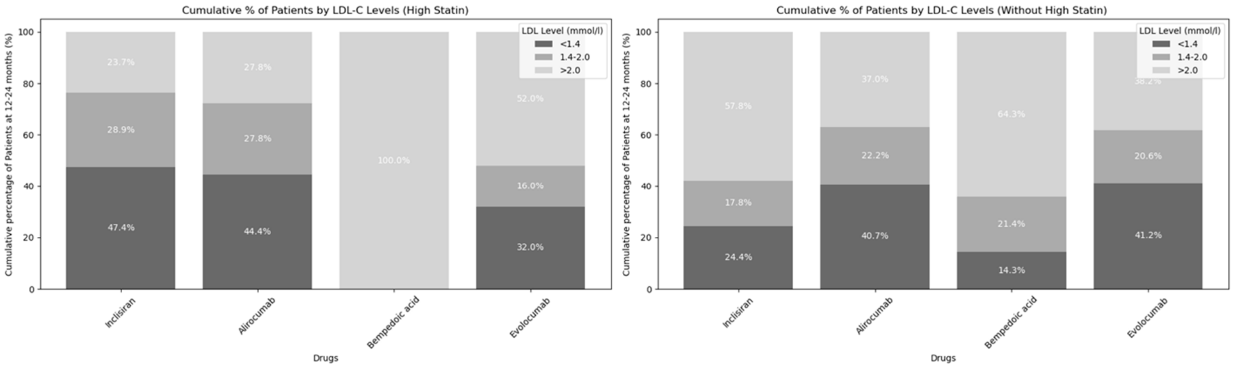

Figure S1: Percentage of patients (with or without high intensity dose statin) reaching LDL-C targets as per NICE guidelines (<2.0 mmol/l) and ESC guidelines (<1.4 mmol/l) during treatment with novel lipid-lowering medications at 12-24 months. Evolocumab and inclisiran appear to be more effective in patients reach guideline recommended lower LDL-C levels, particularly when combined with high intensity statins.

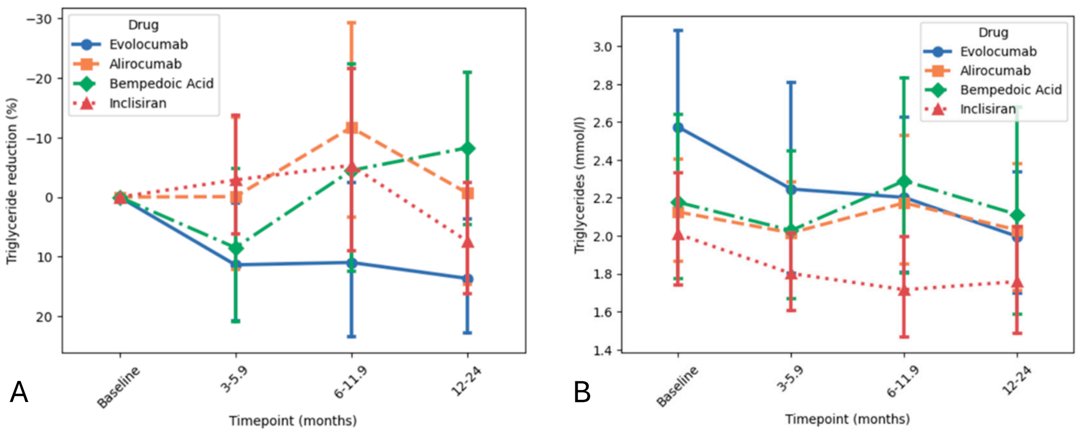

Figure S2: Association of novel lipid lowering medications with reductions in triglycerides at different time points. Panels A and B show the percentage change and the achieved reduction in total triglyceride level over time respectively. The main assessment was the percentage change in triglyceride levels from baseline to 12-24 months, was analysed using Analysis of Variance (ANOVA). P-values for the comparison of drugs for the percentage change were not statistically significant for any of the drugs (P= 0.34). Error bars represent 95% confidence interval around the mean.

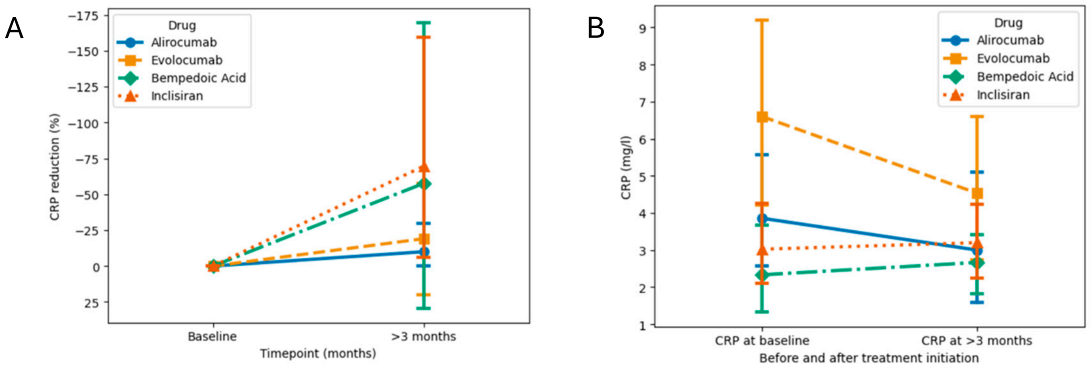

Figure S3: Association of novel lipid-lowering medications with changes in CRP at baseline and >3 months. Panels A and B show the percentage change and the absolute change in CRP level over time respectively. Change in level of CRP from baseline to >3 months, was analysed using ANOVA. P-values for the comparison of drugs for the percentage change were not statistically significant for any of the drugs and B show the absolute change in CRP over time respectively (see Supplementary Table S4). Error bars represent 95% confidence interval around the mean.
